# Patient-reported outcome measures in community mental health teams: pragmatic evaluation of PHQ-9, GAD-7 and SWEMWBS

**DOI:** 10.1192/bjb.2019.20

**Published:** 2019-10

**Authors:** Paul Blenkiron, Lucy Goldsmith

**Affiliations:** 1 Tees, Esk and Wear Valleys NHS Foundation Trust, UK; 2 St George's, University of London, UK

**Keywords:** Community mental health teams, patients, rating scales, outcome studies, out-patient treatment

## Abstract

**Aims and method:**

We evaluated routine use, acceptability and response rates for the Patient Health Questionnaire (PHQ-9), Generalised Anxiety Disorder Scale (GAD-7) and Short Warwick-Edinburgh Mental Well-Being Scale (SWEMWBS) within adult community mental health teams. Measures were repeated 3 months later. Professionals recorded the setting, refusal rates and cluster diagnosis.

**Results:**

A total of 245 patients completed 674 measures, demonstrating good initial return rates (81%), excellent scale completion (98–99%) and infrequent refusal/unsuitability (11%). Only 32 (13%) returned follow-up measures. Significant improvements occurred in functioning (*P* = 0.01), PHQ-9 (*P* = 0.02) and GAD-7 (*P* = 0.003) scores (Cohen's *d* = 0.52–0.77) but not in SWEMWBS (*P* = 0.91) scores. Supercluster A had higher initial PHQ-9 and GAD-7 scores (*P* < 0.001) and lower SWEMWBS scores (*P* = 0.003) than supercluster B. Supercluster C showed the greatest functional impairment (*P* = 0.003).

**Clinical implications:**

PHQ-9 and GAD-7 appear acceptable as patient-reported outcome measures in community mental health team. SWEMWBS seems insensitive to change. National outcome programmes should ensure good follow-up rates.

Reliable, valid and practical outcome measures are a priority for mental health services.^1^ It is now essential for clinical teams to report outcomes in order to evaluate their work, demonstrate effectiveness and support future commissioning decisions.^2^ However, few pragmatic studies exist to inform delivery of mental health outcomes programmes,^3,4^ including current initiatives within the UK National Health Service (NHS).

The NHS quality agenda promotes three central themes: effective services, safety and a positive patient experience.^5^ Arguably, it is the users of services who are best placed to judge how they feel.^6^ Patient-reported outcome measures (PROMs) are standardised questionnaires that elicit subjective reports of health and illness. They aim to assess the personal effects of symptoms, functioning, problems, risks and general well-being on an individual's quality of life. However, no single PROM has evidence of validity across all areas of mental health.^7^ Community mental health teams (CMHTs) are a key component of specialist mental healthcare, yet front-line use of PROMs has not been systematically evaluated in this setting. In addition, it remains unclear how outcomes in secondary care vary across mental healthcare clusters.^8^ ‘Clustering’ is an important tool within the National Tariff Payment System (‘Payment by Results’) and is recommended by NHS England in order to deliver its Five Year Forward View for Mental Health.^9^

## Aims of this study

This study evaluated routine use of three PROMs within adult CMHTs: the Patient Health Questionnaire (PHQ-9), the Generalised Anxiety Disorder Scale (GAD-7) and the Short Warwick-Edinburgh Mental Well-Being Scale (SWEMWBS). We aimed to:

(a) assess completion rates and patient acceptability;
(b) evaluate responsiveness – comparing measures at initial assessment and at review/discharge across mental health superclusters

## Method

This project was registered as a service evaluation by the Department of Research and Development at Leeds and York Partnership NHS Foundation Trust and granted NHS research governance approval (R&D no: LYPFT 2014/498/L).

### Setting and participants

Secondary care mental health services in York and Selby are provided to a population of 280 000 by a specialised mental health trust. The population is predominantly White British (95%), with those of Asian ethnicity (2.2%) representing the largest single ethnic minority.^10^ We collected data from May to October 2014 from the two large ‘ageless’ adult CMHTs. NHS data from trust informatics during the study showed that 38% of contacts were new referrals, with 31% of patients classified as being under the Care Programme Approach (CPA). A mean of 78% of the total caseload were being seen each month.

### Data collection

We included patients aged 18 years and over attending CMHT appointments. Individuals were receiving care from one or more professionals at a psychiatric clinic, at a community mental health team base, at home or in another setting.

Patients were invited to complete the SWEMWBS, PHQ-9 and GAD-7 scales together. Measures were posted with the appointment letter to new referrals to CMHTs, with a request to hand them to the professional they saw. Staff also offered the measures at the initial appointment to individuals who had not completed them. Patients were asked to complete the measures again at follow-up 3 months later, or at discharge if sooner. Follow-up questionnaires were offered in person at the appointment by reception staff or the professional the individual saw. We introduced the study at a team business meeting and obtained staff agreement to participate before the start. In addition to verbal reminders at team meetings during the 6 month study period, we contacted staff individually by e-mail on two occasions (at 3 and 5 months) to remind them to collect follow-up questionnaires.

Patients could choose to complete the measures before, during or immediately after their appointment. Forms explicitly stated that if an individual did not feel like completing the questionnaires, they could decline and this would not affect their care. The questionnaires also informed patients that they could choose to receive this information in audio format (for example, as a CD) or in other languages, including via an interpreter.

Using a standardised *pro forma* attached to the measures, we asked staff to record details about the clinical setting, the reason for seeing the patient, and the main mental health problem (care cluster and diagnosis). To assess return rates accurately, at both initial and follow-up time points we specifically asked staff to return the *pro forma* even if an individual was unable (or declined) to complete measures. Staff also entered the responses into the computerised clinical record. Missing data were later accessed from this record.

### Outcome measures

#### PHQ-9

PHQ-9 is a nine-item measure of depressive symptoms.^11^ Each item is rated using four ordinal response options (0, not at all; 3, nearly every day), giving a severity score between 0 and 27. PHQ-9 also rates difficulty in functioning. A score greater than 9 indicates clinically significant depression. The PHQ-9 is well validated against standard criteria, demonstrates sensitivity to change and is used in a variety of clinical settings.^12,13^

#### GAD-7

GAD-7 is a seven-item measure of anxiety symptoms.^14^ Each item is rated on the same four ordinal responses as the PHQ-9, giving a severity score between 0 and 21. A score above 7 is recommended to identify a likely anxiety disorder.

PHQ-9 and GAD-7 form part of the UK Department of Health's National Minimum Data Set.^3^ Their use is supported by the National Institute for Health and Care Excellence for assessing clinical progress in mental health services.^1^

#### SWEMWBS

SWEMWBS is a short version of a measure originally developed to monitor well-being in the general population and to evaluate policies addressing well-being.^15,16^ There are seven items, each with five response categories (1, none of the time; 5, all of the time). The score range is 7–35 and higher scores indicate greater mental well-being. At the time of this study, the local NHS adopted SWEWWBS within the Regional Care Pathways and Packages Project, designed to implement Mental Health Payment by Results. SWEMWBS has been reported to have adequate internal consistency and reliability.^17^ It has not been systematically evaluated in mental health populations. The developers recommended that sensitivity to change be demonstrated before its introduction into clinical settings.

Prospectively, we also aimed to analyse responses to the following three key questions separately.

• *Self-harm risk (*PHQ-9 question 9): ‘How often have you been bothered by thoughts of being better off dead or of hurting yourself in some way?’ This question is of particular interest in clinical risk assessments.
• *Functional impairment* (additional tenth PHQ-9 question): ‘How difficult have these problems made it for you to do your work, take care of things at home, or get along with other people?’ This question is of practical importance and is independent of symptom scoring.
• *Problem-solving ability* (SWEMWBS question 4): ‘How often over the past 2 weeks would you agree that “I've been dealing with problems well?”’ An inability to solve problems is significantly associated with hopelessness and suicidal intent.^18^

### Data analysis

Data were anonymised and analysed using IBM Statistical Package for Social Sciences for Windows, version 22.^19^ We adjusted total PHQ-9, GAD-7 and SWEMWBS scores for individuals who omitted some replies, using syntax coding with the following formula:
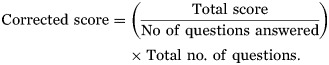


This is a recommended way of handling potential bias in the analysis due to missing items in questionnaires with no subscales.^20,21^ Results are quoted as percentages to the nearest whole number, with totals based on valid known responses. Non-parametric tests were applied to ordinal and continuous variables. We used Spearman's correlation coefficient for independent samples at baseline, Wilcoxon's signed rank (*z*) test for paired data (initial versus final outcomes), and the Kruskal–Wallis *H*-test for differences between superclusters.^22^ Clinical effect sizes (Cohen's *d*) were calculated for reported changes in measures.^23^

## Results

### Response rate

Individuals returned 674 out of 831 questionnaires – a completion rate of 81%. These comprised 277 sets of forms (Table 1). Initial forms were completed by 245 patients, with similar response rates (78–81%) for each PROM. Follow-up forms were received from 32 (13%) individuals. The mean time period between initial and follow-up forms was 74 (s.d. 58) days. There were high rates of scale completion – most respondents answered all questions on each form. Professionals completed their part of the initial forms in 55% (134) cases. Staff recorded that nine (7%) patients declined to fill in the initial forms, and judged it inappropriate to offer forms in another five (4%) cases. No patient was reported to have declined or been judged unsuitable to complete follow-up questionnaires.

**Table 1:** Completion rates for outcome measures

Measure	Initial forms completed (*n* = 245), no. (%)	Final forms completed (*n* = 32), no (%)	No. of questions per form	Total no. questions answered per form, mean (s.d.)
SWEMWBS	192 (78)	26 (81)	7	6.94 (0.31)
PHQ9	198 (81)	31 (97)	9 (+1)	8.88 (0.45)
GAD7	196 (80)	31 (97)	7	6.85 (0.90)

### Patients and setting

The mean age of patients was 47 years (range 18–93), including 127 (60%) women. Initial forms were handed in at a clinic or CMHT base in 99 (74%) cases, and at home or another place in 34 (26%) cases. Most patients (96, 72%) completed forms alone, 16 (12%) received assistance from carers or relatives, and seven (5%) had assistance from staff. No patient asked to receive the questionnaires in an audio format or in another language.

### Professionals

A mean of 27 sets of forms (range 1–50) were returned by 25 professionals. These included five community psychiatric nurses, five social workers, two psychologists, one occupational therapist and 12 psychiatrists (five working age adult consultants, two older age consultants, four core trainees and one higher trainee). Psychiatrists returned twice as many measures per professional (mean 36, 438 forms, 65% of total) as other staff (mean 18, 236 forms, 35%), general *z*-test *P* < 0.0001, 95% confidence interval 61–68%.^20^ The reason recorded for completing initial forms was assessment in 77 (63%) cases, review (including CPA review) in 39 (32%) cases and discharge in three (2%) cases.

### Diagnosis

The main diagnosis according to the 10th edition of the International Classification of Diseases^24^ was available for 211 (86%) of patients. Most common was depressive disorder (acute, recurrent or chronic) in 71 (34%) cases, psychosis (including schizophrenia) in 28 (13%) cases and bipolar disorder (including mania) in 26 (13%) cases. Personality disorder comprised 16 (8%) cases, anxiety disorder 14 (7%), dementia 13 (6%), adjustment disorder 12 (6%), alcohol or drug dependence nine (4%), post-traumatic stress disorder nine (4%), obsessive–compulsive disorder five (2%) and other diagnoses eight (3%).

### Correlations between outcomes

Table 2 describes the associations between measures (construct validity). PHQ-9 and GAD-7 scores were strongly correlated with each other at initial and final appointments. There was a moderate correlation between initial and final PHQ-9 scores, and between initial and final GAD-7 scores. SWEMWBS also showed a moderately strong correlation with concurrent PHQ-9 and GAD-7 scores. However, we found no significant correlation between final SWEMWBS and initial scores on any of the measures.

**Table 2:** Correlations between measures for initial and follow-up appointments

Measure	Initial SWEMWBS	Initial PHQ9	Initial GAD7	Final SWEMWBS	Final PHQ9	Final GAD7
Initial SWEMWBS	–	−0.77***	−0.70***	0.20	−0.45*	−0.48*
Initial PHQ9	−0.77***	–	0.81***	−0.02	0.48**	0.49**
Initial GAD7	−0.70***	0.81***	–	0.09	0.31	0.52**
Final SWEMWBS	0.20	−0.02	0.09	–	−0.56**	−0.49**
Final PHQ9	−0.45*	0.48**	0.31	−0.56**	–	0.83***
Final GAD7	−0.48*	0.49**	0.52**	−0.49**	0.83***	–

Values are Spearman's *r*. *P < 0.05, **P < 0.01, ***P < 0.001.

### Initial versus final scores

Table 3 shows initial and final outcome scores for the paired data (*n* = 32). Applying Wilcoxon's signed rank test, PHQ-9 and GAD-7 scores were significantly lower at review, whereas SWEMWBS showed no significant change. For specific questions, patients' median ratings for thoughts of self-harm and also for their ability to function day to day improved significantly. Respondents' perceived ability to solve problems did not change significantly.

**Table 3:** Initial and final scores for outcome measures

Measure	Initial score, median (IQR)	Final score, median (IQR)	Change in paired scores, median (IQR)	Wilcoxon's (*z*) test *P*-value
SWEMWBS	17.0 (13.0–22.75)	16.5 (13.0–24.25)	−1.5 (−6.25 to +6.3)	0.91
PHQ9	19.6 (11.0–23.0)	12.0 (6.75–20.75)	−3.0 (−9.75 to +3.0)	0.02*
GAD7	15.0 (9.0–18.0)	7.5 (2.75–12.25)	−2.0 (−8.0 to 0.0)	0.003**
Thoughts of self-harm or being better off dead	1.0 (0.0–3.0) On several days in past 2 weeks	(0.0–2.0) Not at all	0.0 (−1.0 to 0.0)	0.008**
Difficulty in functioning day to day	2.0 (1.0–3.0) Extremely difficult	1.0 (0.0–2.25) Somewhat difficult	0.0 (−1.0 to 0.0)	0.013**
Ability to deal with problems	2.0 (2.0–3.0) Rarely	3.0 (1.0–4.0) Some of the time	0.0 (−1.0 to +2.0)	0.80

IQR, interquartile range. *P < 0.05, **P < 0.01.

Text shows wording of median response.

The mean initial PHQ-9 score of 16.8 (s.d. 7.6) decreased on review to 12.6 (s.d. 8.6), representing a moderate effect size (Cohen's *d* = 0.52) across the total sample. The mean initial GAD-7 score of 12.9 (s.d. 6.2) also improved at follow-up to 8.1 (s.d. 6.1), indicating a large effect size (*d* = 0.77).

To examine whether there was any selection bias in follow-up responses, we compared initial median scores for those who did (*n* = 32) and did not (*n* = 213) complete final measures. The Mann–Whitney *U*-test for independent samples showed no significant difference on the SWEMWBS (*P* = 0.91), PHQ-9 (*P* = 0.42) or GAD-7 (*P* = 0.78).

### Age, gender and time interval

For both initial and final measures, older patients answered fewer questions on the PHQ-9 (*r* = −0.52, *P* = 0.002) and GAD-7 (*r* = −0.31, *P* < 0.001). Age was correlated positively with initial SWEMWBS score (Spearman's *r* = 0.36, *P* < 0.001) and negatively with initial PHQ-9 (*r* = −0.15, *P* = 0.04) and ability to function (*r* = −0.17, *P* = 0.03). There was no significant association between age and any final outcome (SWEMWBS, PHQ-9, GAD-7 or functioning). We found no significant association between gender and initial or final measures. The time period between completion of initial and final forms also showed no significant correlation with any initial or final outcome.

### Superclusters

Tables 4 and 5 describe patients and their outcomes across the three supercluster categories.^8^ There were significant differences between PHQ-9, GAD-7 and SWEMWBS scores at initial but not final review. Individuals with non-psychotic disorders (supercluster A) had lower initial SWEMWBS scores (*P* < 0.001), and high levels of anxiety and depressive symptoms that improved at review. Respondents with psychosis (supercluster B) had the lowest PHQ-9 and GAD-7 scores (*P* = 0.003). Those with organic disorders (supercluster C, mainly dementia or cognitive impairment) had the greatest difficulty in functioning (*P* = 0.003) based on the PHQ-9 functioning question). They also reported significant depression, anxiety and self-harm thoughts. Insufficient responses were received to calculate reliable final median outcome scores for supercluster C.

**Table 4:** Mental health superclusters: age, risk, functioning and problem solving, initial responses

Supercluster	No. (%)	Age (years)	Thoughts of self-harm or being better off dead	Difficulty in functioning day to day	Ability to deal with problems
A. Non- Psychotic	144 (68)	45.0	2.0	2.0	2.0
B. Psychotic	54 (26)	44.5	1.0	1.5	3.0
C. Organic	13 (6)	84.0	3.0	3.0	3.0
*P*-value		<0.001***	0.036*	0.003**	0.002**

Figures are corrected median scores.

*P*-values from Kruskal–Wallis *H*-test for differences between superclusters. *P < 0.05, **P < 0.01, ***P < 0.001.

**Table 5:** Mental health superclusters: initial and final outcome scores

Supercluster	Initial SWEMWBS	Initial PHQ9	Initial GAD7	Final SWEMWBS	Final PHQ9	Final GAD7
A. Non- psychotic	15.0	20.125	15.0	17.0	12.0	8.0
B. Psychotic	22.0	11.0	9.0	15.0	11.0	6.0
C. Organic	24.5	24.0	17.0			
*P*-value	<0.001***	0.003**	0.002**	0.48	0.51	0.63

Figures are corrected initial median scores.

*P*-values from Kruskal–Wallis *H*-test for differences between superclusters. **P < 0.01, ***P < 0.001.

## Discussion

This is the first study to examine the pragmatic integration of the PHQ-9, GAD-7 and SWEMWBS within routine CMHT practice. For these three PROMs, we found good initial return rates (80%), excellent rates of scale completion (98–99%) and low rates (11%) of patient refusal or unsuitability. After 3 months, patients reported significant improvements in symptoms of depression and anxiety, self-harm thoughts and functioning, but not in subjective well-being or perceived ability to handle problems.

It is important that outcomes are validated for the population in which they are used. Decreasing anxiety scores were observed across superclusters A and B. Building on research in other settings,^25,26^ our study provides new evidence that the GAD-7, like PHQ-9, is responsive to change in a community mental health population. For depressive symptoms, a drop of more than five PHQ-9 points is reported to indicate a significant and reliable clinical improvement.^27^ We found an eight-point reduction in PHQ-9 scores in supercluster A, which includes those diagnosed with depressive disorder. This effect is similar in size to those observed in large randomised treatment trials for depression.^28^ These findings suggest that both PHQ-9 and GAD-7 might be adopted as PROMs within secondary mental healthcare in functional (non-dementia) populations.

This study has several limitations. Patients and professionals were not asked about their views on the usefulness of collecting these PROMS, or about possible harms. It is also uncertain whether professionals used the responses during their meetings with patients to improve the quality of care (rather than simply to measure it). Furthermore, we do not know the extent to which the improvements observed were due to professional interventions (including medication and psychosocial approaches) rather than the passage of time or regression to the mean.

An important finding is the low collection rate (*n* = 32, 13%) for follow-up measures in ‘real world’ clinical practice. Other mental health outcome studies have also recorded follow-up rates as low as 10–25%, even after (as in our study) professionals are prompted.^29,30^ The difference between initial and final response rates might in part be linked to the number of requests to complete measures. For completion of initial measures, patients were asked both in writing (posted with the appointment letter) and again in person at the appointment. By contrast, collection of follow-up measures relied on staff remembering to ask patients to complete forms at face-to-face clinical review alone.

While the low final response rate limits some conclusions drawn, the outcome score changes observed may be generalisable to the wider patient population for several reasons. First, our analysis comparing initial median scores for completers versus non-completers showed no significant difference in either PHQ-9, GAD-7 or SWEMWBS. Second, response rates by gender ratio were similar at initial and final follow-up, and the time interval between initial and final measures showed no significant relation with any outcome. We have no evidence to support the idea that individuals who improved the most were more, or less, likely to complete final measures. This suggests that attrition bias at follow-up – due to variations between patients in symptoms, functioning or well-being – is less likely. Third, we observed a large (50-fold) variation in the collection of PROMS between professionals. For example, psychiatrists returned twice as many initial and follow-up questionnaires as other team members. In conclusion, it appears more likely that differences in staff engagement with the study, and inconsistent prompting of patients to complete measures (rather than patient characteristics) may account for the variations in return rates.

However, a good response rate remains central to the future success of PROMs.^3,6,9^ This may be improved in busy CMHTs by providing clinicians with adequate administrative time and support, and by implementing robust electronic collection systems.^2,3^ For example, patients could complete forms directly on an electronic tablet linked to their clinical record.

Consistent with previous research, individuals with psychosis rated their well-being on SWEMWBS higher than those with affective disorders.^31^ However, overall SWEMWBS scores did not change in this study, and there was no significant correlation between initial and follow-up SWEMWBS ratings. There are several possible explanations for this. First, subjective well-being could lag behind improvements in symptoms and functioning. Second, SWEMWBS includes questions about areas such as feeling useful and close to people,^16^ which could be measuring something different from other outcomes. Third, the psychometric properties of SWEMWBS may include lower internal reliability and less sensitivity to clinical change than other PROMs. Future research in this population could evaluate the responsiveness of SWEMWBS using methods such as the standardised response mean,^32^ which allows for improvement or worsening over time. Alternative well-being measures are currently being developed. Recovering Quality of Life (http://www.reqol.org.uk) is a new national well-being PROM commissioned by the UK Department of Health. Specifically designed to assess quality of life and recovery outcomes in adults with different mental health conditions, it has been tested in 6000 mental health patients.^30^ The brief version (ReQoL-10) is now freely available for clinical and research use in the UK NHS.

## About the authors

**Paul Blenkiron**, MMedSc, FRCPsych, is a Consultant Psychiatrist in Adult Mental Health at Tees, Esk and Wear Valleys NHS Foundation Trust, and Honorary Professor at Hull York Medical School; **Lucy Goldsmith**, PhD, is a Research Assistant at the Population Health Research Institute, St George's, University of London, UK.
